# Simultaneous Study of Circular RNAs and Messenger RNAs in Colorectal Cancer: The Unbalanced Fate of a Couple?

**DOI:** 10.3390/cancers18030496

**Published:** 2026-02-03

**Authors:** Corentin Levacher, Joanna Delfosse, Camille Charbonnier, Françoise Charbonnier, Mathieu Viennot, Edwige Kasper, Jacques Mauillon, Nathalie Parodi, Stéphanie Baert-Desurmont, Philippe Ruminy, Claude Houdayer

**Affiliations:** 1 Univ Rouen Normandie, Inserm U1245, Normandie Univ, F-76000 Rouen, France; corentin.levacher2@univ-rouen.fr (C.L.); delfossejoanna@gmail.com (J.D.); francoise.charbonnier@inserm.fr (F.C.); 2 Univ Rouen Normandie, Inserm U1245, Normandie Univ, CHU Rouen, Department of Biostatistics, F-76000 Rouen, France; camille.le-clezio@chu-rouen.fr; 3 Univ Rouen Normandie, Inserm U1245, Normandie Univ, Centre Henri Becquerel, F-76000 Rouen, France; mathieu.viennot@chb.unicancer.fr (M.V.); philippe.ruminy@chb.unicancer.fr (P.R.); 4 Univ Rouen Normandie, Inserm U1245, Normandie Univ, CHU Rouen, Department of Genetics, F-76000 Rouen, France; edwige.kasper@chu-rouen.fr (E.K.); s.baert-desurmont@chu-rouen.fr (S.B.-D.); 5 Univ Rouen Normandie, Normandie Univ, CHU Rouen, Department of Genetics, F-76000 Rouen, France; jacques.mauillon@ch-havre.fr (J.M.); nathalie.parodi@chu-rouen.fr (N.P.)

**Keywords:** circular RNAs, messenger RNAs, colorectal cancer

## Abstract

Background/Objectives: Circular RNAs (circRNAs) are emerging players in human diseases, with functions as part of competing endogenous networks. Given the importance of messenger RNA (mRNA) regulation in human diseases and the potential of circRNAs in this regulation, we studied the circRNA–mRNA couple in blood within a cohort of 712 patients suspected of having hereditary colorectal cancer (CRC) and 249 matched controls. Methods: The circRNA–mRNA couple was studied by SEALigHTS (Splice and Expression Analyses by exon Ligation and High-Throughput Sequencing) with a panel of 23 genes involved in CRC predisposition, comprising 788 probes designed at exon ends, enabling the exploration of all exon–exon junctions. Following reverse transcription and probe hybridization on cDNA, nearby probes were ligated, and the number of ligations was quantified using unique molecular identifiers and sequencing. Results: We identified 220 circular junctions, including 47 novel ones. The circRNA/mRNA ratio was 2.42-fold higher in patients compared to controls (*p* < 10^−16^), irrespective of age at cancer onset. This increase was mainly driven by *POLD1* (fold change 3.84) and a single circPOLD1(3,2) with oncogenic potential Conclusions: This study supports the existence of a physiological balance between circRNA and mRNA that can be disrupted under pathological conditions. It rules out a competitive mechanism between circular and linear transcripts in CRC predisposition and raises questions about the role of specific circRNAs in the development of CRC, either as a cause or a consequence.

## Simple Summary

Circular RNAs (circRNAs) can compete with messenger RNAs (mRNAs) from their host gene, which can lead to the downregulation of mRNA. We investigated this potential mechanism of downregulation in colorectal cancer (CRC) predisposition in a large cohort of 712 patients suspected of having hereditary CRC and 249 healthy controls, with a focus on 23 genes associated with CRC. Using the novel SEALigHTS technique (Splice and Expression Analyses by Exon Ligation and High-Throughput Sequencing), we identified 220 circular RNA junctions, including 47 new ones. Patients exhibited a circRNA/mRNA ratio approximately twice that of controls, particularly for the *POLD1* gene, which produces a circRNA with potential cancer-promoting effects. The findings imply that the balance between circRNAs and mRNAs is disturbed in CRC, though not due to competition between the two. They also suggest that specific circRNAs play a role in CRC development, either as a cause or a consequence.

## 1. Introduction

Known for decades [1,2], the messenger RNA (mRNA) is the centerpiece of the transcriptome. This intermediary between DNA and protein is regulated by various mechanisms, including transcription [3,4,5], splicing [6], stability [7], capping [8], transport [9], and translation [10]. Among the elements involved in this regulation, many types of RNA have been documented. MicroRNAs, as well as pseudogene transcripts and long non-coding RNAs, have been shown to play a role in regulating mRNA [11]. These different elements are grouped together in a network known as competing endogenous RNA (ceRNA), and accumulating evidence points to its preponderant role in various pathologies such as cancer [12], neurodegenerative [13], cardiovascular [14], and autoimmune diseases [15]. Recently, with growing interest, circular RNAs (circRNAs) have been included in this ceRNA network [16].

circRNAs are single-stranded, closed-loop covalent structures, devoid of 5′ caps and 3′ poly-A tails. They result from a process known as backsplicing, whereby a 5′ splice site binds to an upstream 3′ splice site [17]. Interest in circRNAs has increased greatly, mainly because the levels of certain circRNAs have proved important for improved diagnosis, prognosis, and disease monitoring in Alzheimer disease [18], cardiovascular disease [19], and cancer [20]. These observations are often attributed to their functions as microRNA and RNA binding protein sponges [21], but other mechanisms are emerging, e.g., cap-independent translation that enables the generation of peptides involved in different tumorigenesis pathways [22,23], or transcription modulation. This last mechanism is particularly interesting, as it has been shown that circRNAs can regulate the levels of various mRNAs, including those of the parental gene [24,25], and a physiological balance between these two forms of transcript has been demonstrated [26,27]. Consequently, it may be hypothesized that a disruption of this physiological balance represents a novel, hidden mechanism in genetic diseases [28,29,30,31]. The question is particularly relevant in diseases where known mechanisms explain only a fraction of cases; the remainder are referred to as “missing heritability”. Colorectal cancer (CRC) exemplifies this issue, as the majority of non-polyposis familial and early-onset microsatellite stable (MSS) cases have no identified genetic cause [32]. To investigate this hypothesis, we performed a simultaneous analysis of circRNA and mRNA expression in blood samples for 23 CRC predisposition genes in a cohort of 712 CRC patients (i.e., the “missing heritability” cohort) and 249 matched controls, using a novel, dedicated technique named SEALigHTS (Splice and Expression Analyses by exon Ligation and High-Throughput Sequencing) [29,33]. We described the backsplicing landscape for these genes, studied the circRNA–mRNA balance between patients and controls, and searched for a competitive mechanism between circRNA and mRNA expression.

## 2. Materials and Methods

**Patients and controls**

The collection of unexplained CRC cases was previously described [32] and includes 1029 patients with a personal and/or familial history suggestive of hereditary predisposition to CRC, with Lynch syndrome and polyposis excluded (Table 1). All patients were recruited by the French network of Cancer Genetics Departments. In addition, 500 healthy volunteers without any personal or familial CRC history among their first-degree relatives were recruited by the Clinical Investigation Center of Rouen University Hospital. Among them, 712 patients and 500 controls had their blood sampled in PAXgene tubes for RNA extraction. To ensure quality, PAXgene tubes were transported within two days after sampling to the investigating center and extracted within 2 weeks. All 712 patients were included in the study. Among 500 healthy controls with available RNA samples, 249 were randomly selected, with a comparable sex distribution and a similar age range to the patient group (Table 2). Written informed consent was obtained from all participants (ethics approval: DC-2013–1759).

Continuous variables are reported as mean ± standard deviation, and categorical variables as number (percentage). Age corresponds to age at blood sampling. RNA concentration refers to total RNA extracted from PAXgene blood samples. Group comparisons were performed using Welch’s *t*-test for continuous variables and the chi-square test for categorical variables.

**RNA extraction**

RNA was extracted from peripheral blood using the PAXgene Blood RNA kit (PreAnalytiX, Switzerland), and concentration was determined with the NanoDrop 1000 Spectrophotometer (ThermoFisher Scientific, MA, USA), with a mean of 88 ng/µL (min: 4; max: 446).

**SEALigHTS**

*Principle*

Initially used as a multiplex technique for fusion transcript detection [34] and for measuring gene expression [35], SEALigHTS has been adapted and validated to study and quantify splicing and backsplicing [29]. Briefly, SEALigHTS allows the simultaneous exploration of all exon–exon junctions in a panel of genes of interest, thanks to probes designed at exon extremities. Following reverse transcription and probe hybridization on cDNA, nearby probes are ligated if splicing and/or backsplicing occurs, and the number of ligations is quantified using unique molecular identifiers and high-throughput sequencing. All possible combinations of exons, i.e., splicing and backsplicing, are detectable.

*Protocol*

Oligonucleotides probes contained (i) specific sequences for each exon (19–30 bases in length to obtain an optimal melting temperature of 70 °C), (ii) Unique Molecular Identifiers (UMI), consisting of 7 random bases, to count the number of ligations, (iii) complementary sequences of universal PCR primers. In accordance with the transcripts present in Ensembl and/or GTex Portal and the literature, 788 probes (Supplementary Table S1) were designed at exon boundaries for the 23 CRC predisposition genes listed by French experts from the Groupe Génétique et Cancer (GGC) [36] (Table 3). For exon ends where a single-nucleotide polymorphism (SNP) is present with a frequency higher than 1% in the Caucasian population, the corresponding probe was designed with a combination of the 2 nucleotides involved to avoid any hybridization problems. If the alternative and canonical splice sites to be explored were close, partial probe overlap occurred. In this case, and when the sequence overlap was greater than 50%, the alternative probe was not selected. To control for DNA contamination, intronic probes were designed for each gene and added to the probe mix (Supplementary Table S1). Following quantification, 14−500 ng of total RNA were converted into cDNA using a SuperScript™ VILO™ cDNA Synthesis kit (Invitrogen, Carlsbad, CA, USA). cDNAs were incubated for 1 h at 60 °C with the mix of 788 oligonucleotide probes (Supplementary Table S1) in 1 × SALSA MLPA buffer (MRC Holland, Amsterdam, The Netherlands). Following hybridization, neighboring probes were ligated using the thermostable SALSA DNA ligase (MRC Holland, Amsterdam, The Netherlands).

Among the 23 genes studied (Table 3), gene expression reported by the GTex Portal varied up to 10,000 times higher for some genes than for others. After an initial trial, it appeared that the high expression of *PTEN* and *RPS20* required a separate amplification mix in combination with the Q5^®^ Hot Start High-Fidelity 2X Master Mix (NEB, Ipswich, MA, USA), which is why 2 independent PCRs were performed using different universal PCR primers. PCR products were purified using AMPure XP magnetic beads (Beckman Coulter, Brea, CA, USA). The library containing *PTEN* and *RPS20* was diluted 1:20 in the library containing the other genes. Sequencing of amplicons was carried out using a NextSeq^®^ system with 75 cycles (Illumina, San Diego, CA, USA).

Sequencing reads, comprising a maximum of 75 bases, including UMIs, left and right probes, and barcodes, were demultiplexed using the barcodes and aligned with probe sequences. Exon junctions were counted for quantification purposes, with the 7 random bases of the UMI allowing 16,384 different combinations of unique molecules. A minimum of 65% mapping of left and right probes was deemed necessary to avoid primer dimers and ensure sufficient ligation and reliable results. A custom Python (v3.11) script, available on request, generated schematic backsplicing and splicing profiles (Supplementary Figure S1).

**Bioinformatics and statistical analyses**

*Ratio circRNAs/mRNAs*

Linear mRNAs (splicing) were distinguished from circRNAs (backsplicing) based on the order of the probes. If a probe located at the 3′ boundary of an exon is ligated with a probe located at the 5′ boundary of the following exon, the transcript is linear. Conversely, if this 3′ probe is ligated to a 5′ probe of a preceding exon, the transcript is circular (Supplementary Figure S1). Each circular RNA is a unique molecule, so all circular junctions must be considered, unlike an mRNA molecule composed of successive linear junctions. For each gene, the ratio between circRNAs and mRNAs was obtained by dividing the total number of UMI counts of all circular junctions by the median number of UMI counts of all canonical linear junctions of that gene, i.e., $\frac{UMI\;all\;circular\;junctions}{median\;UMI\;all\;canonical\;linear\;junctions}\times 100$. Importantly, some UMIs attributed to linear junctions may actually derive from circRNAs. This occurs when a circRNA contains multiple exons, allowing probes to hybridize to internal exons. Consequently, the signal may be misinterpreted as linear junctions, affecting mRNA expression calculation. To mitigate this, only linear junction UMIs with no potential influence from circRNAs expressed at more than 10% of the corresponding mRNA were counted for mRNA calculation. A Wilcoxon rank-sum test was used to assess differences between patient and control groups, and *p*-values were corrected using the Bonferroni method to control the overall type I error rate at 5%.

*mRNA and circRNAs expression*

For each sample and junction, the UMI counts for each junction were normalized according to library size, i.e., $\frac{UMI\;junction}{UMI\;all\;junctions}=UMI\;normalized\;junction$. For each gene and sample, mRNA expression was calculated as the mean of normalized “circular-free” canonical linear junctions. In contrast, as circRNAs represent distinct transcripts, expression per gene was obtained by summing the UMIs of normalized circular junctions.

**Circular RNAs characterization**

To characterize the full circRNA sequences, divergent primers were designed (Supplementary Figure S2A,B). From 500 ng of RNA, cDNA was generated using the SuperScript™ VILO™ cDNA Synthesis kit (Invitrogen, Carlsbad, CA, USA), followed by PCR with the Q5^®^ Hot Start High-Fidelity 2X Master Mix kit (NEB, Ipswich, MA, USA). Gel-purified PCR products were sequenced using PCR primers and the BigDye v3 kit (Applied Biosystems, South San Francisco, CA, USA) and analyzed by capillary electrophoresis on a “3500 Genetic Analyzer” (Applied Biosystems, South San Francisco, CA, USA).

## 3. Results

### 3.1. General Considerations

Of the 961 samples analyzed, 657 (68.4%), i.e., 433 patients and 224 controls, met the quality criteria (65% mapping), with a mean UMI count of 1,183,735, ensuring homogeneous samples and results, as shown by principal component analysis (PCA) (Supplementary Figure S3 and Supplementary Table S2).

*GREM1* and *EPCAM* genes are poorly expressed in whole blood. As the number of UMIs is gene expression-dependent, we were unable to detect all canonical junctions; therefore, *GREM1* and *EPCAM* were excluded from further analyses.

**Figure 1:**
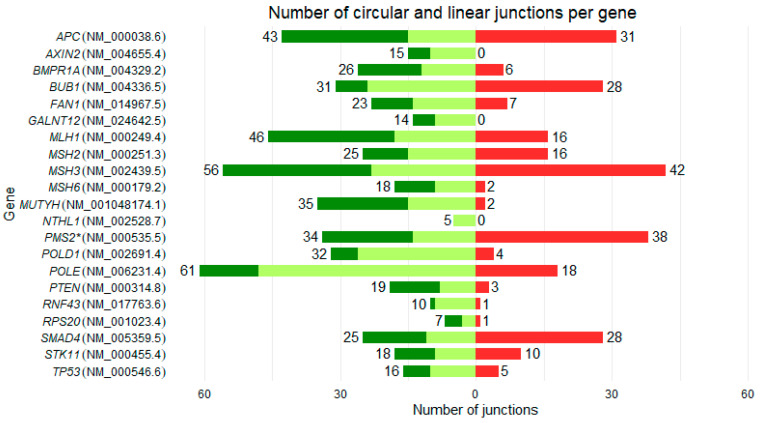
Number of circular and linear junctions per gene. Canonical (NM references) and alternative junctions are indicated in light green and green, respectively. Circular junctions are indicated in red. The number of linear and circular junctions detected is shown in front of the bars. *PMS2* is indicated with an asterisk due to probable detection of pseudogene junctions.

For the 21 genes analyzed, 559 linear junctions (canonical and alternative) were identified (Supplementary Table S1), with diversity in their numbers between genes (Figure 1 and Table 3). Although the number of linear junctions depends on the number of exons, the number of expressed alternative junctions differed between genes, e.g., *APC* and *MSH2* both have 16 exons, but 43 and 25 total linear junctions were expressed in blood, respectively. As the probes hybridize on both *PMS2* and its pseudogene *PMS2CL*, their respective mRNAs and circRNAs cannot be distinguished. Therefore, *PMS2* was not considered in the analyses.

### 3.2. Backsplicing Landscape

Of the 20 genes studied (*PMS2* excluded), SEALigHTS detected 220 circular junctions (Supplementary Table S1), of which 47 (21.36%) were novel (Table 3), not reported in RJunBase [37], a database compiling data on linear junctions, circular junctions, and fusion transcripts. A wide diversity in the number and expression level of circRNAs (Table 3 and Figure 1) was observed. Three genes (*AXIN2*, *GALNT12,* and *NTHL1*) did not show circular junctions. For the remaining 17, the median number of circular junctions per gene was 7 and ranged from 1 to 42 for *RPS20* and *MSH3*, respectively. The number of circular junctions was not linked to the number of linear junctions; e.g., *MUTYH* had 35 linear junctions and only two circular junctions, unlike *SMAD4,* which had 25 and 28 respectively (Table 3 and Figure 1). While circRNAs were expressed at low levels for most genes (17/20), *POLD1, BUB1,* and *SMAD4* had circRNA levels similar or higher than their mRNA molecules. The relative expression of circRNA to mRNA varied between genes and was not related to the number of circRNAs produced by the gene. For example, *POLD1* had the highest circRNA/mRNA expression level at 731.23%, i.e., a relative abundance of 731 circRNAs per 100 mRNA molecules, with only four circRNAs produced, whereas *APC* had a ratio of 12.19% with 31 circRNAs produced (Table 3).

**Table 3:** Splicing and backsplicing summary for each gene. The number of exons is indicated according to the reference transcript. The number of linear junctions includes canonical and alternative ones. The number of new circular RNAs is indicated with RJunBase as reference. *PMS2* is indicated with an asterisk due to pseudogene issues (see text). *GREM1* and *EPCAM* genes are not included in the table, as they were excluded from the analysis due to lack of expression. Genes are listed by the ratio of circular to linear UMIs. Values above 100% were obtained since mRNA expression is estimated from the median of multiple canonical linear junctions per transcript, while each circRNA is counted through a single backsplice junction (see Section 2).

Gene	Reference Transcript	Number of Exons	Number of Linear Junctions	Number of Circular Junctions	Number of New Circular RNAs	Circular/Linear Junctions (%)	Circular/Linear UMIs (%)
POLD1	NM_002691.4	27	32	4	3	12.50	731.23
BUB1	NM_004336.5	25	31	28	8	90.32	492.85
PMS2 *	NM_000535.7	15	34	38	17	111.76	145.69
SMAD4	NM_005359.6	12	25	28	6	112.00	114.25
POLE	NM_006231.4	49	61	18	6	29.51	61.42
MSH3	NM_002439.5	24	56	42	1	75.00	39.01
RNF43	NM_017763.6	10	10	1	0	10.00	23.69
FAN1	NM_014967.5	15	23	7	0	30.43	20.81
MLH1	NM_000249.4	19	46	16	6	34.78	19.67
MSH2	NM_000251.3	16	25	16	2	64.00	15.95
APC	NM_000038.5	16	43	31	3	72.09	12.19
MUTYH	NM_001048174.2	16	35	2	1	5.71	4.05
STK11	NM_000455.5	10	18	10	4	55.56	2.93
BMPR1A	NM_004329.3	13	26	6	2	23.08	2.28
MSH6	NM_000179.3	10	18	2	2	11.11	2.24
TP53	NM_000546.6	11	16	5	2	31.25	2.15
PTEN	NM_000314.8	9	19	3	0	15.79	0.18
RPS20	NM_001023.4	4	7	1	1	14.29	0.04
AXIN2	NM_004655.4	11	15	0	0	0.00	0.00
GALNT12	NM_024642.5	10	14	0	0	0.00	0.00
NTHL1	NM_002528.7	6	5	0	0	0.00	0.00

* PMS2 is indicated with an asterisk due to pseudogene issues.

Following circRNA detection, we aimed to describe the entire sequence of the most frequent circRNAs (Supplementary Table S3), i.e., circPOLD1(3-2), circBUB1(19-10), circBUB1(9-2), circBUB1(9-6), circSMAD4(8-6), circSMAD4(10-5), and circSMAD4(8-5), and found alternative backsplicing events in two cases. Sequencing the circRNA joining exons 3 to 2 of *POLD1* (circPOLD1(3-2)) characterized a circPOLD1(3-2p) (Supplementary Figure S2C), i.e., with use of an alternative splice site in exon 2, reported in RJunBase for both linear and circular transcripts. For the circRNA joining exons 10 to 5 of *SMAD4* (circSMAD4(10-5)), sequence analysis revealed a full-length backsplice transcript and an alternative one with exons 6 and 7 skipped (Supplementary Figure S2D). As these alternative splicing events are also present in mRNAs, this suggests that the same (back)splicing machinery produces both messenger and circular RNAs, increasing circRNA diversity.

### 3.3. Ratio circRNA/mRNA Between CRC Patients and Controls

**Figure 2:**
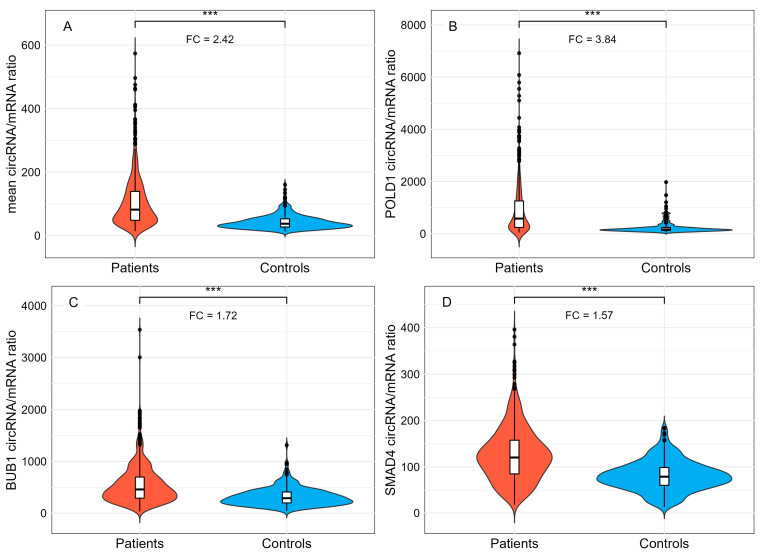
Ratio circRNA/mRNA between CRC patients and controls. (**A**) Mean ratio for the 17 genes is indicated for patients and controls in red and blue violin plots, respectively. Mean ratio between patients and controls for (**B**) *POLD1*, (**C**) *BUB1*, and (**D**) *SMAD4* violin plots. Fold Changes (FC) were calculated using the mean ratios of patients and controls. (***: *p* < 0.001; Wilcoxon test). Black dots represent outliers (mean +/−2DS).

The ratio was calculated for the 17 genes harboring circular junctions (Supplementary Table S2). We observed a significant 2.42-fold increase in the mean circRNA/mRNA ratio in patients compared to controls (105 vs. 43; (Wilcoxon test, *p* < 10^−16^), (Figure 2A and Supplementary Figure S4). This result was confirmed independently for *POLD1*, *BUB1,* and *SMAD4*, which exhibited the highest circRNA/mRNA ratios. Hierarchical clustering confirmed these findings and did not reveal confounding factors (Supplementary Figure S5). The increase in the circRNA/mRNA ratio was predominantly driven by *POLD1* (Figure 2B), with a fold change of 3.84, rather than by *BUB1* and *SMAD4* (fold changes of 1.72 and 1.57, respectively) (Figure 2C,D). We identified 31 outlier patients, i.e., patients with a circRNA/mRNA ratio above mean +/−2 DS. Among these patients, 11 exhibited outlier ratios for all three genes (*POLD1*, *BUB1*, and *SMAD4*), while 12 and 8 patients displayed outlier ratios for two and one of these genes, respectively. To explore potential genotype–phenotype correlations, we investigated whether this subgroup of 31 patients was associated with specific clinical phenotypes. No significant association was found between this subgroup and the presence of adenocarcinoma (*p* = 0.43; Fisher’s exact test), adenoma (*p* = 0.75; Pearson’s chi-squared test), familial cancer (*p* = 0.51; Pearson’s chi-squared test), early sporadic cancer (*p* = 0.41; Fisher’s exact test), or multiple primary tumors (*p* = 0.64; Fisher’s exact test). Age and sex were tested and did not appear to be confounding factors (*t*-test *p*-value = 0.887 and Pearson’s chi-squared test: *p*-value: 0.55, respectively).

**Figure 3:**
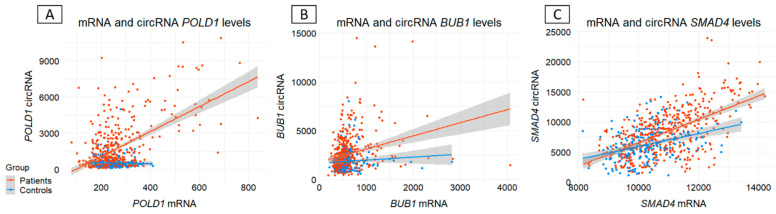
Linear regression analysis between circular RNAs (circRNAs) and messenger RNAs (mRNAs) levels for (**A**) *POLD1*, (**B**) *BUB1,* and (**C**) *SMAD4*. X-axis: mRNA expression; Y-axis: circRNA expression. mRNA expression was calculated by averaging the unique molecular identifiers (UMIs) of canonical junctions. circRNA expression corresponds to the sum of circular junction UMI counts. Orange and blue points represent patients and controls, respectively. Linear regression was performed on all samples and is shown by lines of the same color, with the area of uncertainty shown in gray.

To shed light on a possible mechanism, we then investigated whether this circRNA/mRNA increase was linked to a decrease or increase in mRNA or circRNA levels, respectively. Regression analysis between circRNA and mRNA expression showed that this imbalance probably reflected increased circRNA production or accumulation rather than decreased *POLD1*, *BUB1,* and *SMAD4* expression, thereby suggesting independent regulation (Figure 3).

## 4. Discussion

CircRNAs are now established as key players in human pathology, but original data remain scarce. We investigated whether disruption to the coregulation of mRNA–circRNA could represent a novel disease mechanism in a cohort of 712 unexplained CRC cases and 249 matched controls. While our data do not support a competitive regulatory mechanism, they reveal a significant imbalance in the mRNA–circRNA couple in blood between CRC patients and controls.

These results were obtained using SEALigHTS, a novel targeted high-throughput approach that, to our knowledge, is the only technology enabling the simultaneous quantitative analysis of mRNAs and circRNAs, including from FFPE-compatible material. By relying on probe hybridization at exon boundaries and UMI-based quantification prior to amplification, SEALigHTS circumvents the major limitations of conventional RNA-seq strategies for circRNA detection [38], without requiring RNA enrichment or specific sample treatment [39].

Our study provides new insights into (i) the mechanism of backsplicing, (ii) the landscape of circRNAs for 20 CRC genes, and (iii) the involvement of circRNAs from these 20 genes in CRC. These different aspects are discussed successively. Linear splicing and backsplicing of exons are mediated by the spliceosome and occur simultaneously during the transcription of most human genes. Briefly, two main models for exonic circRNA biogenesis coexist. In the first model, known as “lariat-driven circularization” or “exon skipping”, skipping of one or more exons from the pre-mRNA results in a spliced mRNA and a lariat containing the exon(s) that will be circularized. In the second model, “intron-pairing-driven circularization” [17], the presence of intronic repeated inverted complementary sequences, such as Alu sequences, enables the pairing of two introns that border exons that will be circularized. These sequences promote the spatial proximity of the 5′ splice site junction to the upstream 3′ site [40], leading to the formation of a circular transcript. Although both models may explain our results, we counted 220 circular junctions and 138 alternative linear splicing junctions (i.e., exon skipping), which for the most part were not related to circRNAs. For example, *BUB1* has 28 circular junctions but only seven alternative linear splicing junctions. Consequently, based on these observations, the “intron-pairing-driven circularization” model is more likely to be involved.

Albeit counterintuitive, the number of circRNAs does not increase with the number of exons or introns (Table 3). This might be explained by independent regulation and/or by the fact that circRNA biogenesis depends on several parameters, such as the size of flanking introns and the presence of Alu sequences (see the “intron-pairing-driven circularization” model), transcription speed [41], notably through interaction with DNA forming circRNA:DNA hybrids (circR loops) [42], and splice site strength [26]. circ*POLD1*(3-2), circ*BUB1*(9-2), and circ*BUB1*(4-3) were more highly expressed than their parental mRNAs, but the vast majority of circRNAs (217/220, 94%) represented less than 10% of the corresponding mRNA (Supplementary Table S1).

These results confirm that backsplicing is less efficient than splicing [43] and suggest a transcriptional backsplicing background, as already described for linear splicing. More surprisingly, at the gene level, overall circRNA expression relative to the corresponding mRNAs is often greater than 10% (9/20 genes; Table 3). The values observed for *POLD1*, *BUB1,* and *SMAD4*, five times higher in the patients’ outlier group for *POLD1* (Figure 2), can hardly be considered background noise and may have biological relevance (Table 3). This suggests that each gene has its own circRNA repertoire, with different and finely regulated but uncoupled circRNA/mRNA expression levels [44,45]. Although a competitive mechanism has been ruled out, we demonstrate that this couple may be unbalanced in certain pathological contexts [20,31]. Few large, high-throughput studies have reported circRNA/mRNA ratios, and, to our knowledge, none have done so in blood. Recently, a large integrative RNA-sequencing analysis of circRNA profiles in colorectal cancer demonstrated that circRNA downregulation was associated with decreased expression of circRNA host genes [46]. We also observed a similar trend in circRNA and mRNA levels, but in the direction of increased expression.

Patients suspected of hereditary predisposition to colorectal cancer exhibited a 2.42-fold higher circRNA/mRNA ratio compared to controls, an imbalance largely driven by *POLD1,* with a 3.84-fold change. No correlation with phenotype was identified for the subgroup of outlier patients with the highest circRNA/mRNA ratios. Our results suggest increased production or impaired clearance of *POLD1* circRNA rather than reduced *POLD1* expression, consistent with the known contribution of *POLD1* to hereditary CRC predisposition, which is related not to decreased expression but to missense pathogenic variants in the exonuclease domain [47]. Interestingly, circPOLD1(3,2) has recently been shown to play a functional role in tumorigenesis. In a recent study, circPOLD1(3,2) expression increased with lesion severity in cervical cancer and promoted oncogenic signaling through interactions with RNA-binding proteins such as YBX1, leading to activation of pathways involved in cell proliferation and tumor progression [48]. These findings support the biological relevance of circPOLD1(3,2) and reinforce the significance of the imbalance observed in our study.

Overall, the circRNA/mRNA imbalance observed in CRC patients suggests that the circRNAs studied play a role in a subset of CRC cases, either as a cause or a consequence of other underlying defects. CircRNAs are known to be finely regulated in a cell-type dependent manner [49]; for example, they are highly expressed in muscle, and tumor studies have shown that circRNA levels are lower in tumors than in surrounding healthy tissue [50]. Thus, the observed imbalance may also reflect changes in cellular composition following tumorigenesis or chemotherapy and may serve as a biomarker associated with cancer onset and progression [20]. Given the phenotype of the cohort studied, i.e., patients suspected of hereditary predisposition to colon cancer, the small subgroup of 31 outlier patients is of particular interest and warrants further investigation, including cosegregation analyses in families, circRNA–mRNA tumor analyses, and the search for newly formed peptides to elucidate novel mechanisms.

## 5. Conclusions

Overall, splicing and backsplicing analyses using SEALigHTS highlight the RNA architecture of individual genes and provide no evidence for a competitive mechanism or coordinated regulation between circular and linear transcripts. The observed imbalance is largely driven by a single circRNA with oncogenic potential (circPOLD1(3,2)), suggesting that specific circRNAs may play a pivotal role in disease susceptibility. These findings open new avenues for investigating circRNAs in blood as potential biomarkers or functional contributors to cancer predisposition.

## Figures and Tables

**Table 1:** Description of the criteria used to select patients for inclusion in the collection.

**Inclusion criteria**	CRC in two first-degree relatives, one being diagnosed before 61 years of age; CRC diagnosed before 51 years of age or advanced colorectal adenoma (diameter over 1 cm, and/or tubulovillous or villous, and/or with high-grade dysplasia) before 41 years of age; Multiple primary CRCs in the same individual, with the first one being diagnosed before 61 years of age.
**Exclusion criteria**	Lynch syndrome, as defined by the presence of a germline MMR gene pathogenic variant and/or an MSI tumor, in the context of a suggestive presentation (familial history, early age of onset); Adenomatous polyposis, as defined by more than 10 histologically proven adeno-mas; Hamartomatous polyposis, as defined by the presence of histologically proven hamartomas.

**Table 2:** Main characteristics of the study cohort.

Characteristic	Patients (*n* = 712)	Controls (*n* = 249)	*p*-Value
Female, *n* (%)	434 (61.0%)	146 (58.6%)	0.59
Male, *n* (%)	278 (39.0%)	103 (41.4%)	
Age at blood sampling, years (±SD)	47.8 ± 9.5	40.5 ± 14.9	<0.001
RNA concentration, ng/µL (±SD)	86.7 ± 44.7	91.5 ± 23.8	0.31

## Data Availability

The data presented in this study are available from the corresponding author upon request.

## Supplementary Materials

The following supporting information can be downloaded at: https://www.mdpi.com/article/10.3390/cancers18030496/s1, Figure S1. Schematic representation of splicing and backsplicing counts. Figure S2. Characterization of circular RNAs (circRNAs) by RT-PCR and Sanger sequencing. Figure S3. Principal Component Analysis (PCA) plot demonstrating the homogeneity of results. Figure S4. Correlation between the mean circRNA/mRNA ratio and age. Figure S5. Heatmap of log-transformed circRNA/mRNA ratios. Table S1. Probe names and sequences listed in alphabetical order. Table S2. Master count table for calculations. Table S3. Mean normalized expression of circular RNAs in patients and controls.
