# Supplementary material for: Simultaneous Study of Circular RNAs and Messenger RNAs in Colorectal Cancer: The Unbalanced Fate of a Couple?
Source: Cancers (Basel). 2026 Feb 3;18(3):496. doi: 10.3390/cancers18030496 (PMC12897111; doi:10.3390/cancers18030496)
Supplement: Supplementary file 1 [file cancers-18-00496-s001.zip › Levacher et al. Revised Supplementary Figures and legends.pdf]

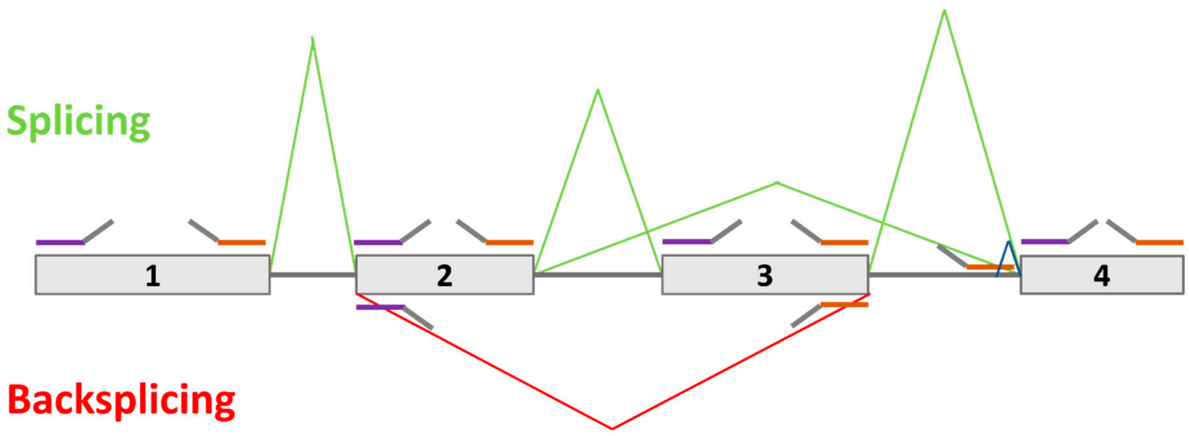

Supplementary Figure S1. Schematic representation of splicing and backsplicing counts. Exons, represented by rectangles, are numerated 1 to 4 and introns by lines. The order of the probes (positioned at the ends of exons) enabled us to differentiate splicing and backsplicing. Green and red peaks represent, respectively linear and back-splicing. Using an intronic probe, the level of contaminating DNA is assessed and represented by the blue peak.

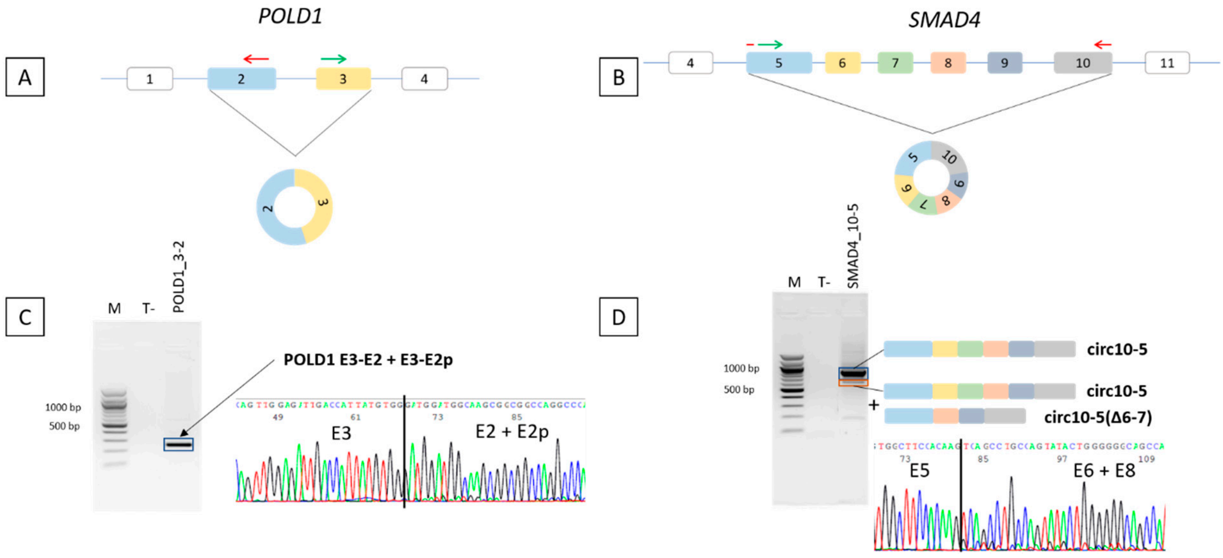

Supplementary Figure S2. Characterization of circular RNAs (circRNAs) by RT-PCR and Sanger sequencing. Schematic experiment design with divergent primers to characterize the full sequence of *POLD1*_circRNA_3-2 (A) and *SMAD4* _circRNA_10-5 (B). Gel migration of products and sequences of corresponding circRNAs revealed alternative splicing for *POLD1* (C) and *SMAD4* (D). (M: size marker; T-: Negative control; bp: base pair)

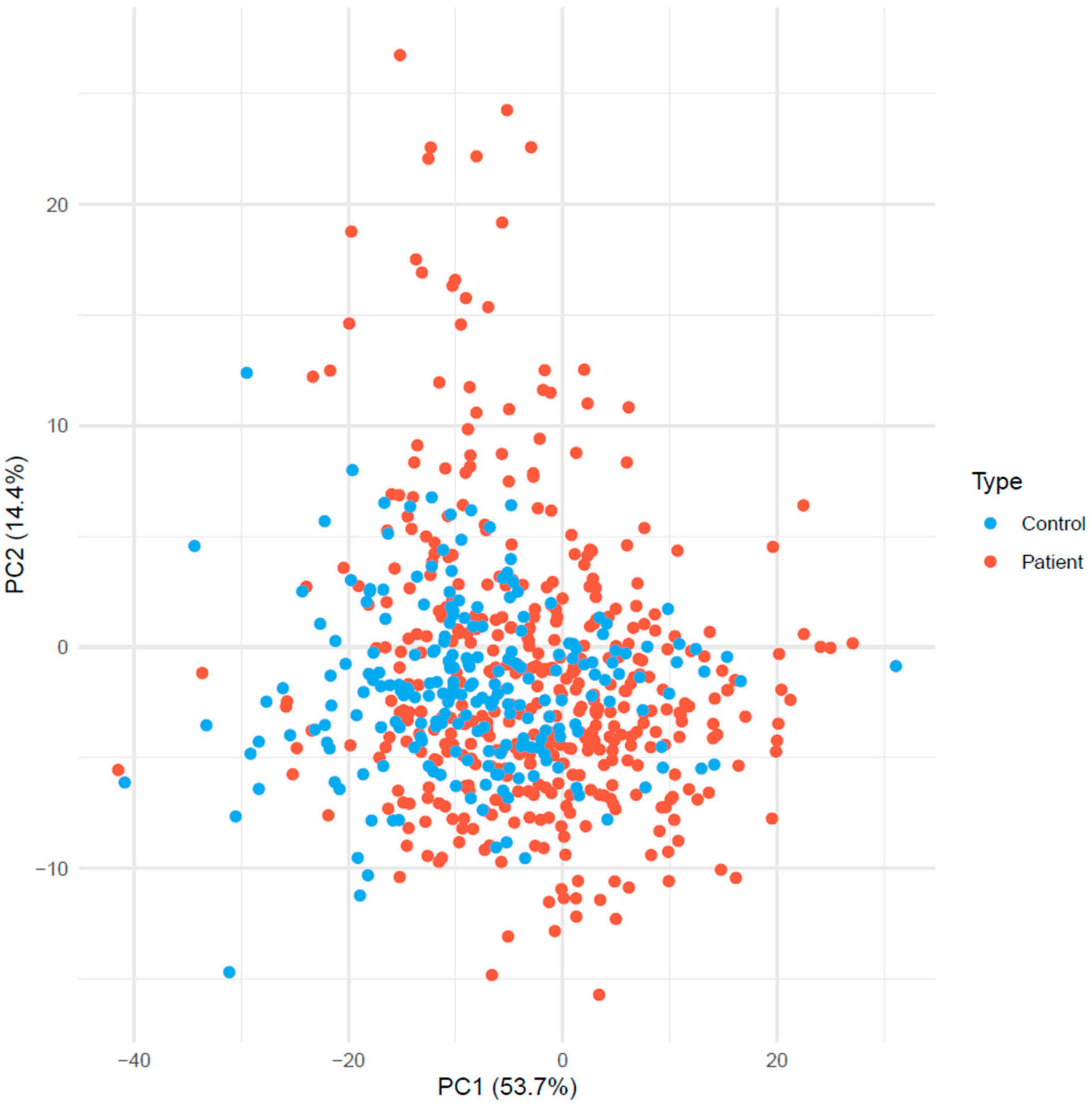

Supplementary Figure S3. Principal Component Analysis (PCA) plot demonstrating the homogeneity of results. Each point represents a sample.

Supplementary Figure S4. Correlation between mean CircRNA/mRNA ratio and age. A statistically significant but weak correlation was observed in the overall cohort (Pearson r = 0.14), consistent with a marginal age-related effect. Importantly, this association was not robust when analyzing patients separately and remained of very small effect size in all analyses (r < 0.2). These results indicate that age may contribute minimally to circRNA/mRNA variability but does not explain the circRNA upregulation observed in the patient subgroup.

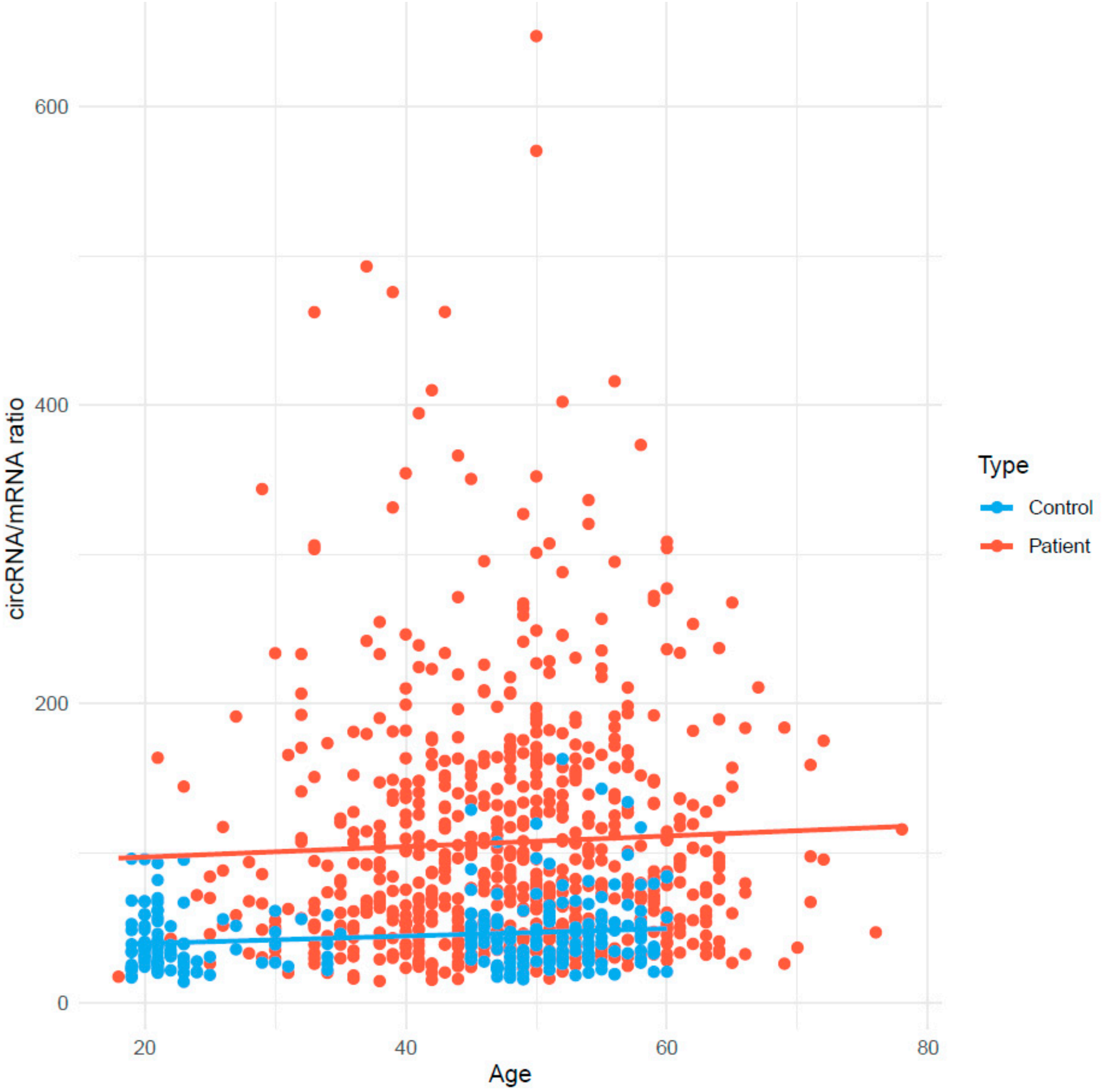

Supplementary Figure S5. Heatmap of log-transformed circRNA/mRNA ratios. Each column correspond to a sample and each row to a gene under study. Samples are annotated by RNA concentration, age, type (patient or control) and sex.

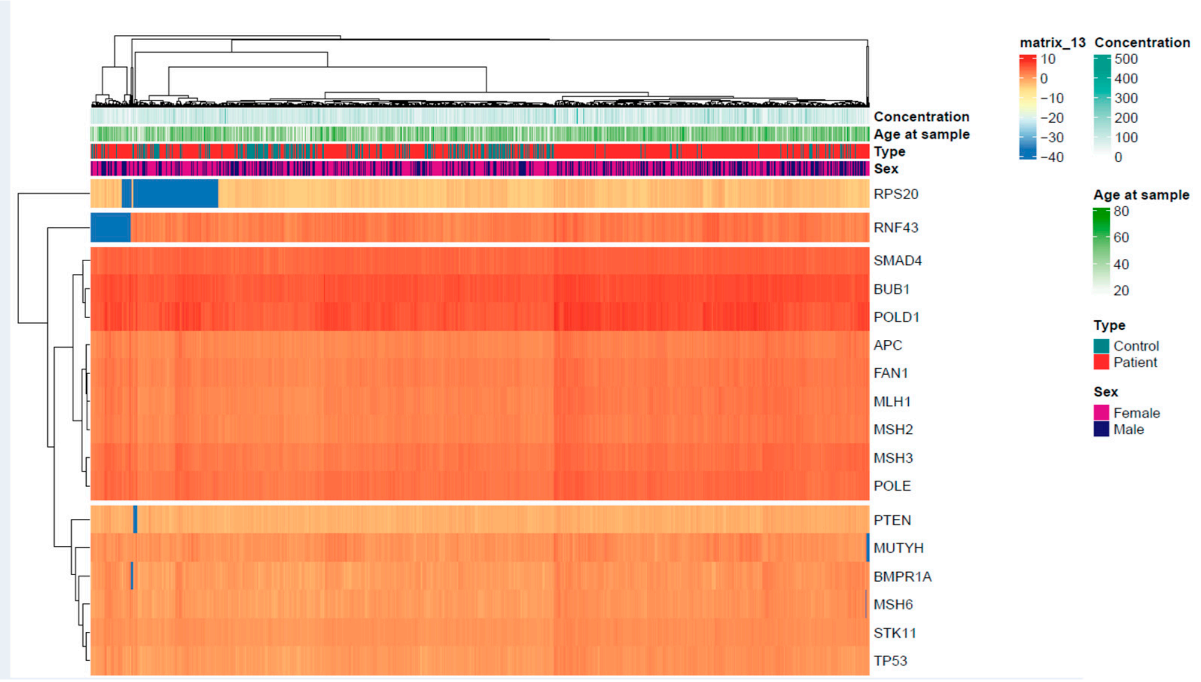

Supplementary Table S1. Probes name and sequences are listed by alphabetic order. All the physiological junctions are listed in the table, with the proportion of detection in all the sample and the type (canonical, exon skipping, alternative or circular) junction information.

Supplementary Table S2. Master count table for calculations.

Supplementary Table S3. Mean normalized expression of circular RNAs in patients and controls.

For each circRNA, expression levels were normalized to the total number of unique molecular identifiers per sample. Values are reported as mean ± standard deviation for patients and controls separately. Circular RNAs are named using the format circGENE(exonA, exonB) to indicate the back-splice junction.
