# Supplementary figures and images for: Simultaneous Study of Circular RNAs and Messenger RNAs in Colorectal Cancer: The Unbalanced Fate of a Couple?
Source: Cancers (Basel). 2026 Feb 3;18(3):496. doi: 10.3390/cancers18030496 (PMC12897111; doi:10.3390/cancers18030496)

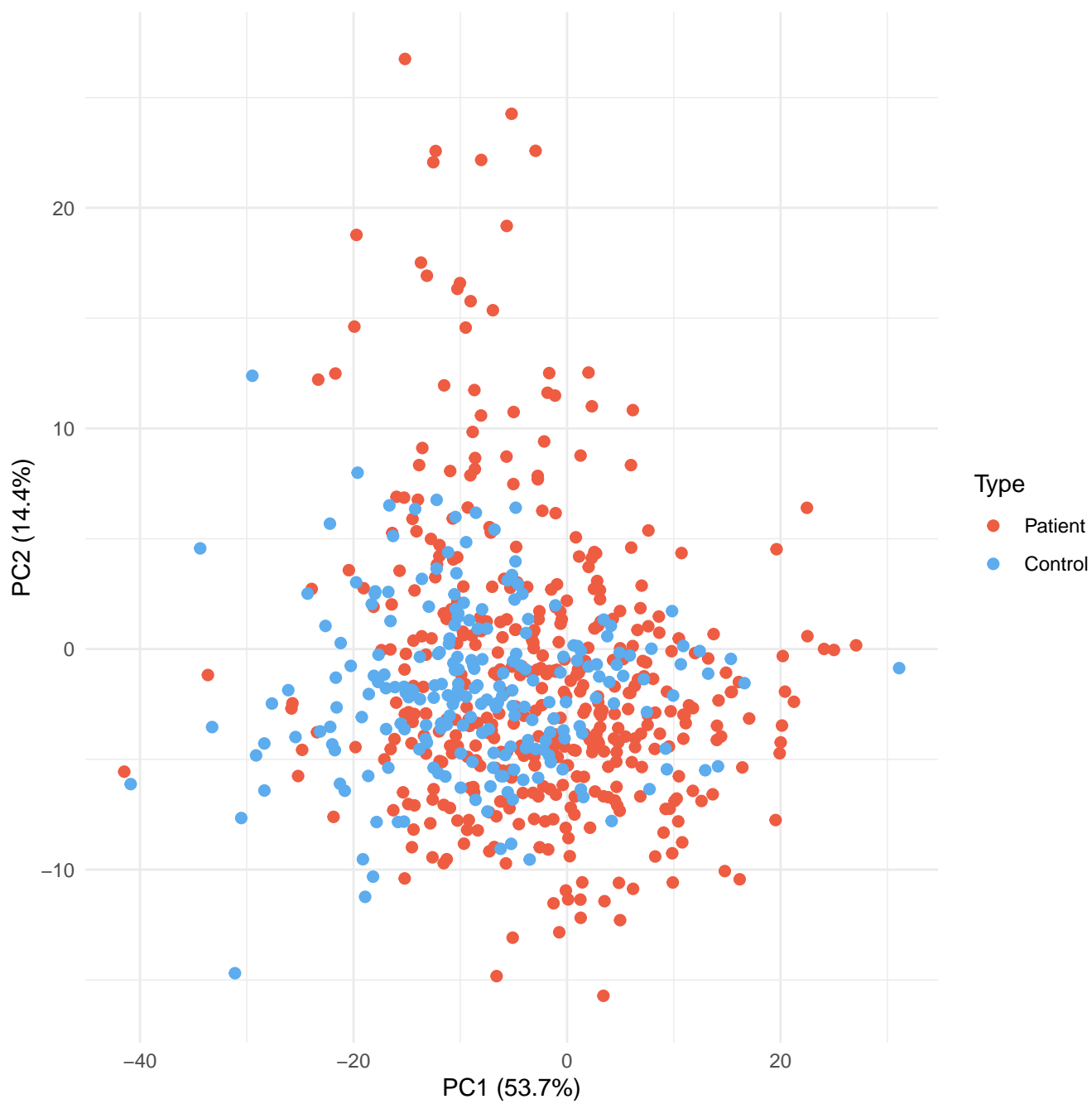

Supplement: Supplementary file 1 [file cancers-18-00496-s001.zip › Levacher et al. Supplementary Figure 3 (modified).pdf]

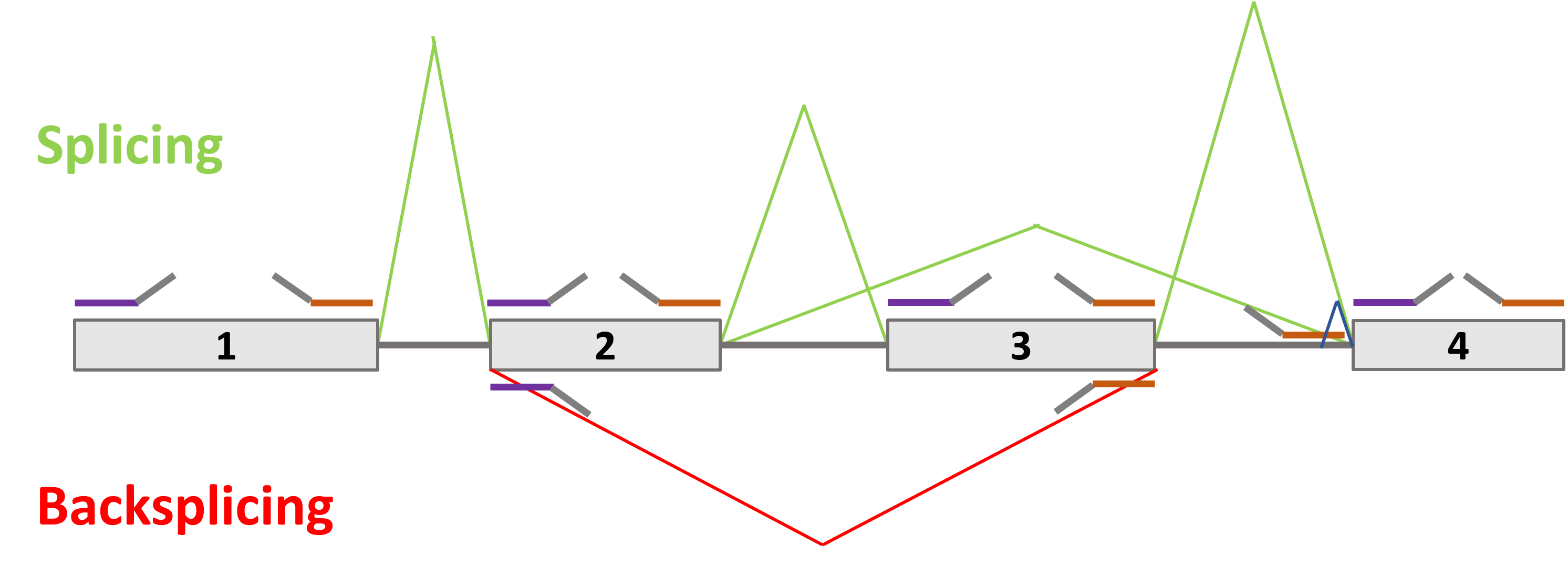

Supplement: Supplementary file 1 [file cancers-18-00496-s001.zip › Levacher et al. Supplementary Figure S1.tif]

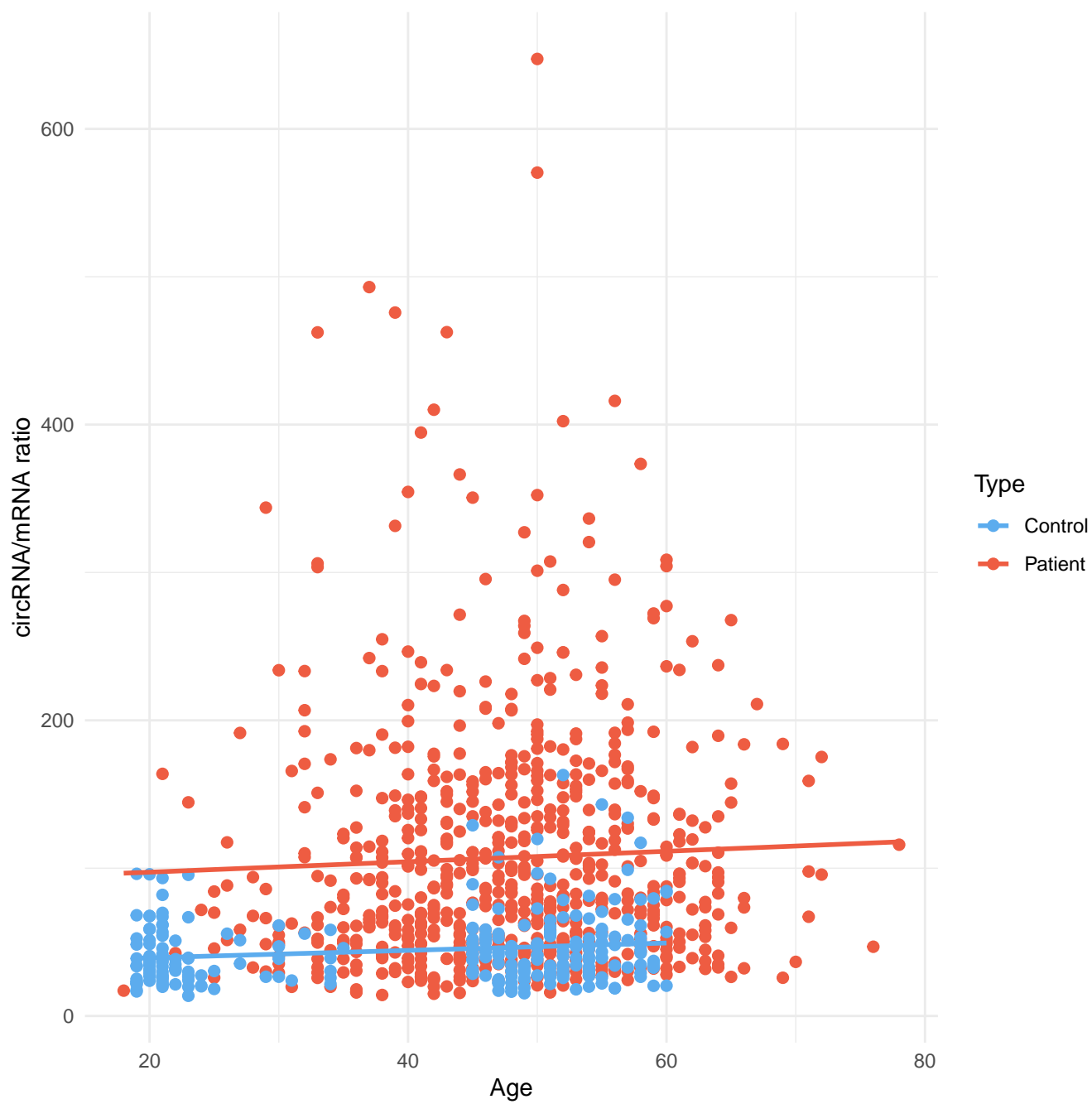

Supplement: Supplementary file 1 [file cancers-18-00496-s001.zip › Levacher et al.Supplementary Figure 4 (new).pdf]

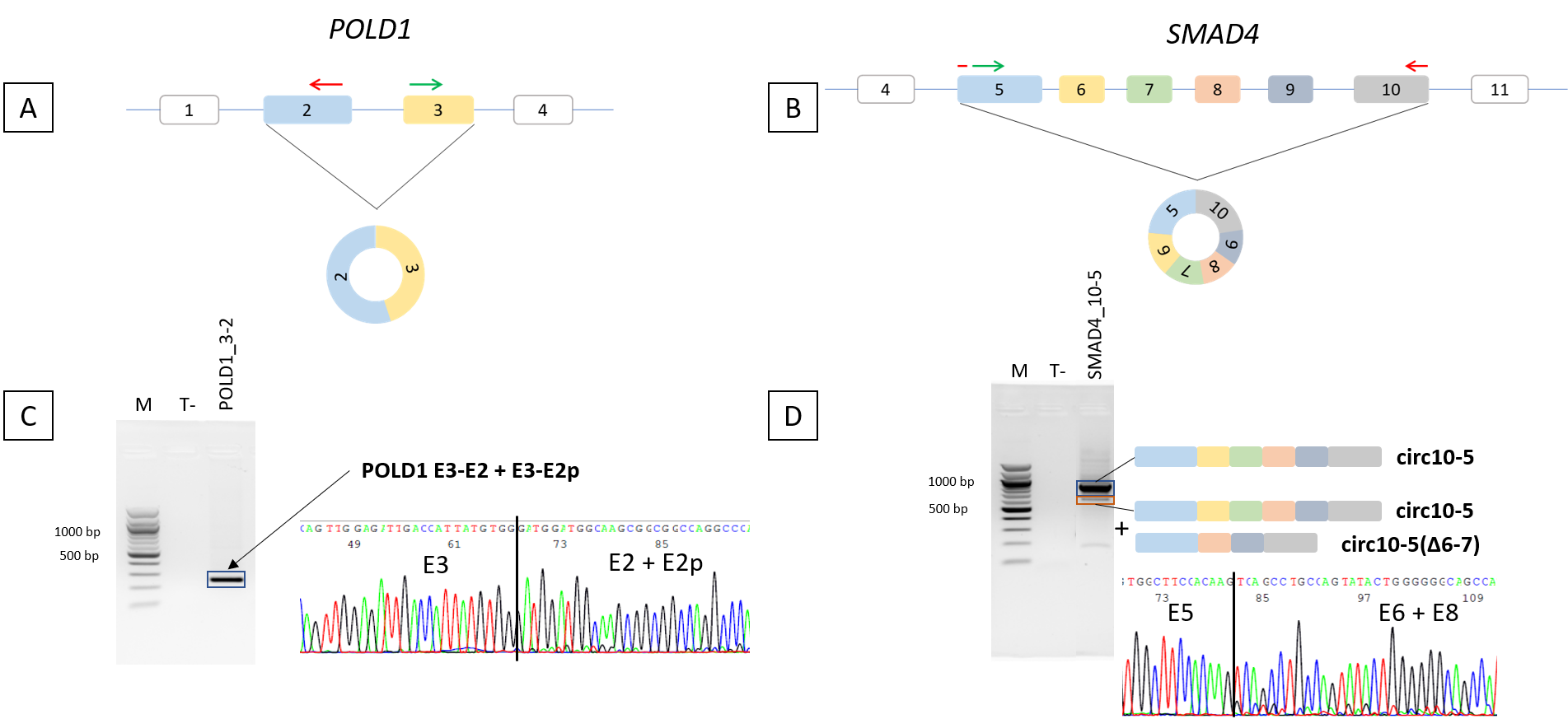

Supplement: Supplementary file 1 [file cancers-18-00496-s001.zip › Levacher et al.Supplementary Figure S2.tif]
